# A Library of Analytic Indicators to Evaluate Effective Engagement with Consumer mHealth Apps for Chronic Conditions: Scoping Review

**DOI:** 10.2196/11941

**Published:** 2019-01-18

**Authors:** Quynh Pham, Gary Graham, Carme Carrion, Plinio P Morita, Emily Seto, Jennifer N Stinson, Joseph A Cafazzo

**Affiliations:** 1 Institute of Health Policy, Management and Evaluation Dalla Lana School of Public Health University of Toronto Toronto, ON Canada; 2 Centre for Global eHealth Innovation Techna Institute University Health Network Toronto, ON Canada; 3 eHealth Center Universitat Oberta de Catalunya Catalonia Spain; 4 eHealth Lab Research Group School of Health Sciences Universitat Oberta de Catalunya Catalonia Spain; 5 School of Public Health and Health Systems Faculty of Applied Health Sciences University of Waterloo Toronto, ON Canada; 6 Child Health Evaluative Sciences Research Institute The Hospital for Sick Children Toronto, ON Canada; 7 Department of Anesthesia and Pain Medicine The Hospital for Sick Children Toronto, ON Canada; 8 Lawrence S Bloomberg Faculty of Nursing University of Toronto Toronto, ON Canada; 9 Institute of Biomaterials and Biomedical Engineering Faculty of Applied Science and Engineering University of Toronto Toronto, ON Canada

**Keywords:** analytics, effective engagement, engagement, adherence, log data, mobile health, mobile applications, chronic disease, scoping review

## Abstract

**Background:**

There is mixed evidence to support current ambitions for mobile health (mHealth) apps to improve chronic health and well-being. One proposed explanation for this variable effect is that users do not engage with apps as intended. The application of analytics, defined as the use of data to generate new insights, is an emerging approach to study and interpret engagement with mHealth interventions.

**Objective:**

This study aimed to consolidate how analytic indicators of engagement have previously been applied across clinical and technological contexts, to inform how they might be optimally applied in future evaluations.

**Methods:**

We conducted a scoping review to catalog the range of analytic indicators being used in evaluations of consumer mHealth apps for chronic conditions. We categorized studies according to app structure and application of engagement data and calculated descriptive data for each category. Chi-square and Fisher exact tests of independence were applied to calculate differences between coded variables.

**Results:**

A total of 41 studies met our inclusion criteria. The average mHealth evaluation included for review was a two-group pretest-posttest randomized controlled trial of a hybrid-structured app for mental health self-management, had 103 participants, lasted 5 months, did not provide access to health care provider services, measured 3 analytic indicators of engagement, segmented users based on engagement data, applied engagement data for descriptive analyses, and did not report on attrition. Across the reviewed studies, engagement was measured using the following 7 analytic indicators: the number of measures recorded (76%, 31/41), the frequency of interactions logged (73%, 30/41), the number of features accessed (49%, 20/41), the number of log-ins or sessions logged (46%, 19/41), the number of modules or lessons started or completed (29%, 12/41), time spent engaging with the app (27%, 11/41), and the number or content of pages accessed (17%, 7/41). Engagement with unstructured apps was mostly measured by the number of features accessed (8/10, *P*=.04), and engagement with hybrid apps was mostly measured by the number of measures recorded (21/24, *P*=.03). A total of 24 studies presented, described, or summarized the data generated from applying analytic indicators to measure engagement. The remaining 17 studies used or planned to use these data to infer a relationship between engagement patterns and intended outcomes.

**Conclusions:**

Although researchers measured on average 3 indicators in a single study, the majority reported findings descriptively and did not further investigate how engagement with an app contributed to its impact on health and well-being. Researchers are gaining nuanced insights into engagement but are not yet characterizing effective engagement for improved outcomes. Raising the standard of mHealth app efficacy through measuring analytic indicators of engagement may enable greater confidence in the causal impact of apps on improved chronic health and well-being.

## Introduction

### Background

There is mixed evidence to support current ambitions for mobile health (mHealth) apps to improve chronic health and well-being [1]. While some apps have demonstrated efficacy in definitive trials [2-5], others have performed poorly [6-9]. One proposed explanation for this variable effect is that users do not engage with apps as intended [10]. The construct of engagement has been quantitatively conceptualized as the amount, duration, breadth, and depth of intervention usage [11,12]. For many mHealth app evaluations, users can be segmented along a continuum of engagement; some will never use the app, some will use it but quickly abandon it, and some will use it in unexpected ways. Complex patterns of engagement with mHealth apps are emerging and challenge current conceptual paradigms for interpreting their impact on chronic health outcomes. These digitally mediated mechanisms of action require more granular evaluations capable of analyzing multilevel, temporally dense engagement data [13]. Evaluating engagement is therefore a priority and calls for the integration of nonintrusive measures of this construct in mHealth evaluation methodology [14].

Recently, scholars sought to further the conceptualization of engagement by proposing that it may be more valuable to identify the mechanisms that underlie *effective engagement*, defined as *sufficient engagement with an intervention to achieve intended outcomes* [14,15]. The construct of effective engagement differs conceptually from both engagement and adherence, which have historically been used interchangeably [16]. Sieverink et al reason that the following 3 elements are necessary to determine adherence to a digital health intervention: (1) the ability to measure usage behaviors, (2) an operationalization of intended use, and (3) an empirical, theoretical, or rational justification of intended use [17]. We propose that effective engagement is more intentional than engagement but less justified than adherence. It sits between both constructs and bridges the transition from identifying patterns of engagement toward evidencing their capacity to achieve intended outcomes.

There has been recognition that the definition of engagement has evolved to include *offline* interactions with the behavior change mediated by a digital health intervention. Yardley et al have been instrumental in furthering this conceptualization of engagement by suggesting that there are 2 levels of engagement: (1) the *micro* level of immediate engagement with the digital health intervention and (2) the *macro* level of engagement with the wider intervention-mediated behavior change [14]. They posit that engagement is a dynamic process marked by shifts in both micro and macroengagement, which will vary depending on the intervention, the user, and their context. Users may be macroengaging and experiencing positive behavior change, but this may not necessarily be reflected in their microengagement analytics data. In acknowledgment of this distinction between engagement with the technological and behavioral aspects of an intervention, Yardley et al critically posit that microengagement alone cannot be taken as a valid indicator of effective engagement. We do not dispute Yardley et al’s arguments and recognize the limitations of relying solely on microengagement data to infer effective engagement. However, we posit that measuring and reporting on microengagement is fundamental to understanding how people actually use an app to improve their health and well-being. In turn, these analytic insights can be coupled with measures of macroengagement to identify the mediating mechanisms that motivate effective engagement.

The application of analytics, defined as the use of data to generate new insights [18], is an emerging approach to study and interpret engagement with mHealth interventions [19]. Van Gemert-Pijnen et al have advanced the application of log data analysis to inform how an intervention works in practice and which components should be improved to yield greater benefit [20-22]. Arden-Close et al have developed and implemented a novel R-based tool to visually explore patterns of engagement [23]. Heckler et al have called for the adoption of a continuous optimization model of evaluation that leverages simulated computational models to predict how users might engage with an intervention before data collection [24]. Scherer et al have demonstrated the value of joint models in the analysis of longitudinal engagement data. In fact, Scherer et al recently participated in a workshop sponsored by the National Institutes of Health on emerging technology and data analytics for behavioral health, and espoused the need for new analytic methods that can scale to thousands of individuals and billions of data points [19]. Short et al recently published a viewpoint on engagement measurement options that can be employed in electronic health (eHealth) and mHealth behavior change intervention evaluations [25]. They found that system engagement data are the most commonly collected and reported measures of engagement in eHealth and mHealth interventions. From this, they recommend having shared ways of conceptualizing these data as the field progresses to consolidate categorization.

### Objectives

Motivated by the proven value of analytics to study engagement with mHealth apps, we sought to compile and catalog a library of analytic indicators of engagement with consumer mHealth apps for self-managing chronic conditions. We defined analytic indicators as proxy measures of engagement with an mHealth app based on objective usage that generates log data [14,22]. When positioned alongside other measures suitable for evaluating the subjective experience of mHealth app engagement, they may provide complementary data-driven insights into the objective extent of engagement. We propose that analytic indicators of engagement do exactly this: they *indicate* that users may be engaging effectively with a digital health intervention but do not definitively confirm a relationship between engagement and intended outcomes. Establishing this relationship requires adopting a mixed-methods multidimensional approach to measure effective engagement using multiple assessment strategies [14,25].

While many researchers have included analytic indicators as a study measure when evaluating apps, they are not consistent or systematic in their selection [26]. We propose that there is benefit to understanding how engagement with mHealth apps for chronic conditions has been defined, measured, and analyzed across evaluations. The aim of this scoping review was therefore to consolidate how analytic indicators of engagement have previously been applied across clinical and technological contexts to inform how they might be optimally applied in future evaluations.

## Methods

### Review Framework

This scoping review was guided by the methodological framework developed by Arksey and O’Malley [27] and advanced by Levac et al [28]. They endorse an iterative review process with 5 distinct steps: (1) identifying the research question, (2) searching for relevant studies, (3) selecting studies, (4) charting the data, and (5) collating, summarizing, and reporting results. This framework is particularly relevant to disciplines with emerging evidence, such as mHealth, in which the paucity of definitive research makes it difficult for researchers to undertake systematic reviews [28]. In this context, conducting a scoping review allowed us to incorporate a range of study designs beyond those accepted for inclusion in systematic reviews, to generate broad findings on how researchers are measuring engagement with consumer mHealth apps for chronic conditions. We made efforts to adhere to recommendations for each step, starting with the selection of a research question that was sufficiently broad to map the extent, range, and nature of mHealth engagement research activity. We conducted this review to explore the following research question: *what analytic indicators of engagement are being used in evaluations of consumer mHealth apps for chronic conditions?*

### Search Strategy

A literature search was conducted in the MEDLINE, PsycINFO, CINAHL, and EMBASE databases. In addition, the *Journal of Medical Internet Research* and its sister journals were independently searched given their frequent and high-impact publication of mHealth research. A combination of different keywords for the constructs “engagement” and “mHealth” was used. No search terms for chronic conditions were defined a priori to broaden search results. We adopted the World Health Organization’s definition of a chronic condition as a “non-communicable disease of long duration and slow progression [29].” Multimedia Appendix 1 presents our search strategy for MEDLINE on the Ovid platform.

### Eligibility Criteria

Titles and abstracts retrieved from the search strategy were screened for inclusion against the following criteria: (1) the article described an evaluation or a protocol for an evaluation of a consumer mHealth app for self-managing a chronic condition; (2) the study included operationalization of an engagement-related construct—Multimedia Appendix 1 provides the full list of screened constructs; (3) the study included objective, quantifiable measurements using log data analytics; (4) the app was intended to be used more than once; (5) the article was published between November 1, 2015, and November 1, 2017; and (6) the article was published in English.

Studies were excluded if (1) the mHealth app was solely an appointment reminder service; (2) the primary app technology was short message service or interactive voice response; (3) the app was for an acute condition or preventive health purposes; (4) the app was a support tool for a patient’s circle of care; (5) the app did not require user input through active or passive (sensor) data entry; (6) the app only delivered educational content; and (7) the article primarily described the design, development, or usability testing of the app.

### Data Collection and Analysis

The first author conducted the electronic searches with support from a faculty-affiliated librarian and reviewed the reference lists of relevant articles. All identified titles and abstracts were downloaded and merged using Mendeley (Elsevier) [30] and duplicated records were removed. The first author independently screened all titles and abstracts against eligibility criteria. Any articles that caused the author uncertainty were retained until data extraction when more information was available to make an informed decision for inclusion in the review. Following title and abstract review, full papers of included abstracts were assessed for final selection by all study authors.

**Codes extracted from included articles.**

1. General information regarding the study title, authors, journal, year, and country.
2. App information, specifically the public name, chronic condition addressed, and accessibility of health care provider services.
3. Study information, specifically the purpose, duration, sample size, and design.
4. App structure (structured, hybrid, or unstructured): “Structured” apps contained locked, sequential components (eg, modules, lessons, and features) that users had to complete before moving forward. “Hybrid” apps contained both fixed core components and variable components for free use. “Unstructured” apps contained variable components that users could access and use at will.
5. Analytic indicators used to measure engagement, specifically the number of log-ins or sessions logged, the number of modules or lessons started or completed, the number of features accessed, the number of measures recorded, the number or content of pages accessed, the frequency of interactions logged, and total time spent engaging with the app.
6. Engagement-based segmentation: studies that segmented users based on engagement data (eg, “of the users who logged in at least five times…”) were assigned this code.
7. Application of engagement data (descriptive or inferential): a “descriptive” code was assigned to studies that presented, described, or summarized engagement data. An “inferential” code was assigned to studies that used engagement data to predict the intended outcome. Outcome types were coded for studies that applied engagement data inferentially.
8. Attrition type (dropout or nonusage) and statistical method of analysis: dropout attrition is the phenomenon of users not returning to complete follow-up study activities. Nonusage attrition is the phenomenon of users losing interest in a digital health intervention and ceasing to use it [10].

A data extraction form was developed by the first author to extract relevant study information. We referenced work by Sieverink [17] and Kelders [31] on analytic indicators of adherence to eHealth technologies to establish preliminary codes. The form was piloted on a sample of included articles to validate proposed codes and add emergent codes. The codes extracted from each study are presented in Textbox 1. All study data were entered into SPSS version 24 (IBM) [32]. Each study along with its corresponding data was treated as a separate case. We categorized studies according to app structure and application of engagement data and calculated descriptive data for each category. Chi-square and Fisher exact tests of independence were applied to calculate differences between coded variables. A Monte Carlo correction was applied when observed counts were below expected counts.

## Results

### Study Selection

**Figure 1:**
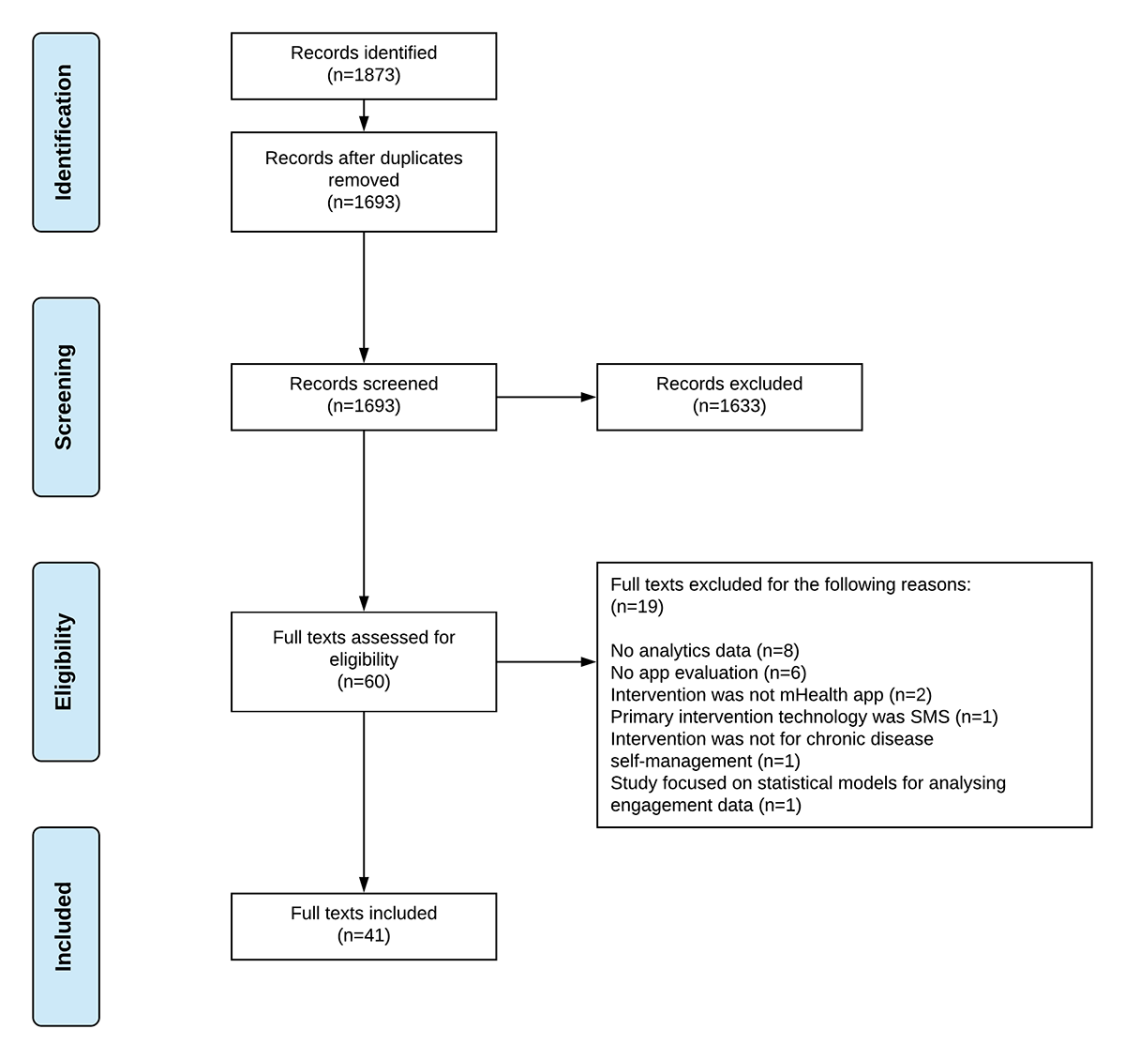
Preferred Reporting Items for Systematic Reviews and Meta-Analyses flow diagram. mHealth: mobile health; SMS: short message service.

A total of 1873 articles were identified through the database search. Of the 60 full texts screened, 19 were excluded, 8 of which did not include objective, quantifiable measurements using log data analytics. In total, 41 articles comprising 33 studies and 8 protocols met the eligibility criteria and were included for review. Figure 1 presents the Preferred Reporting Items for Systematic Reviews and Meta-Analyses flow diagram of the study selection progress [33].

### Methodological Characteristics

The first authors of reviewed studies were affiliated with institutions in the United States (46%, 19/41), Canada (20%, 8/41), the United Kingdom (10%, 4/41), Australia (5%, 2/41), Germany (5%, 2/41), the Netherlands (5%, 2/41), France (2%, 1/41), India (2%, 1/41), Singapore (2%, 1/41), Spain (2%, 1/41), Sweden (2%, 1/41), and Switzerland (2%, 1/41).

Researchers reported log data analytics across 14 different engagement-related constructs: engagement (27%, 11/41), adherence (17%, 7/41), usage (15%, 6/41), use (15%, 6/41), feasibility (10%, 4/41), acceptability (7%, 3/41), utilization (5%, 2/41), attrition (5%, 2/41), participation (5%, 2/41), activity (2%, 1/41), adoption (2%, 1/41), compliance (2%, 1/41), fidelity (2%, 1/41), and retention (2%, 1/41). There was significant variation in how constructs were defined across studies, which limited our ability to (1) extract reliable definitions for each construct, (2) map analytic indicators to specific constructs, and (3) conduct cross-construct comparisons of analytic indicators.

The majority of reviewed studies were experimental (51%, 21/41), with the two-group pretest-posttest randomized controlled trial (RCT) as the most prevalent experimental study design (48%, 10/21), followed by the one-group pretest-posttest design (43%, 9/21). Quasi-experimental design selection (17%, 7/41) was more diverse and included cohort (29%, 2/7), interrupted time-series (14%, 1/7), and single case (14%, 1/7) studies. The remaining 13 studies included for review were observational in design (32%, 13/41). Studies were on average 5 months long (median 152 days, interquartile range, IQR 106), with a sample size of over 100 participants (median 103, IQR 252). The longest reviewed observational study conducted by Serrano et al was 7 years long with over 1 million participants [34]. A total of 19 studies applied engagement-based segmentation and reported results for separate user cohorts (58%, 19/33). In total, 14 of the reviewed studies were published in the *Journal of Medical Internet Research* or its sister journals (34%, 14/41).

### Intervention Characteristics

A wide range of chronic conditions were targeted through the apps under study, with mental health (29%, 12/41), chronic pain (12%, 5/41), asthma (10%, 4/41), cardiovascular disease (7%, 3/41), and diabetes (type 1 and 2; 15%, 6/41) leading the clinical charge. Researchers also evaluated apps for cancer (5%, 2/41), hypertension (5%, 2/41), obesity (5%, 2/41), chronic kidney disease (2%, 1/41), chronic obstructive pulmonary disease (2%, 1/41), cystic fibrosis and inflammatory bowel disease (2%, 1/41), Parkinson disease (2%, 1/41), and sleep apnea (2%, 1/41). Over half of the apps had a hybrid structure (59%, 24/41), 10 apps were unstructured (24%), and 7 apps were structured (17%). Nearly half of all structured apps were aimed at improving mental health (40%, 4/10). Health care provider services were accessible to users to support managing their condition in nearly half of all reviewed apps (44%, 18/41). Characteristics of the included studies are presented in Multimedia Appendix 2 alongside the full dataset of coded analytic indicators for each study, which are summarized below.

### Analytic Indicators

Across the reviewed studies, engagement was measured using the following 7 analytic indicators in order of prevalence: the number of measures recorded (76%, 31/41), the frequency of interactions logged (73%, 30/41), the number of features accessed (49%, 20/41), the number of log-ins or sessions logged (46%, 19/41), the number of modules or lessons started or completed (29%, 12/41), time spent engaging with the app (27%, 11/41), and the number or content of pages accessed (17%, 7/41). Table 1 presents a tally of the analytic indicators measured in each included study. On average, researchers applied 3 different analytic indicators to measure their engagement data (mean 3.20, SD 1.42; median 3, IQR 2). The Fisher exact test of independence indicated that engagement with unstructured apps was mostly measured by the number of features accessed (8/10, *P*=.04), and engagement with hybrid apps was mostly measured by the number of measures recorded (21/24, *P*=.03). Table 2 provides a descriptive overview of structured, hybrid, and unstructured apps across study characteristics and analytic indicators.

**Table 1:** Tally of analytic indicators used in reviewed studies.

Author		Measures	Interactions	Features	Log-ins	Modules	Time spent	Pages
**Mental health (n=12)**								
	Beiwinkel et al [35]	✓^a^	✓	—^b^	✓	✓	✓	—
	Ben**-**Zeev et al [36]	✓	✓	✓	✓	✓	—	—
	Ben**-**Zeev et al [37]	✓	✓	✓	—	✓	—	—
	Davies et al [38]	✓	✓	✓	✓	—	✓	✓
	Frisbee et al [39]	—	—	✓	✓	—	—	—
	Kinderman et al [40]	✓	—	—	✓	—	—	—
	Kuhn et al [41]	—	✓	—	—	—	—	✓
	Owen et al [42]	—	✓	✓	✓	—	✓	✓
	Pham et al [43]	—	✓	—	✓	—	✓	—
	Torous et al [44]	✓	✓	✓	—	—	—	—
	Vansimaeys et al [45]	✓	✓	—	—	✓	—	—
	Wahle et al [46]	✓	✓	—	✓	✓	—	—
**Chronic pain (n=5)**								
	Fortier et al [47]	✓	—	—	—	—	—	—
	Jamison et al [48]	✓	✓	—	✓	—	—	—
	Jibb et al [49]	✓	✓	—	—	—	—	—
	Reade et al [50]	✓	✓	—	✓	✓	—	—
	Skrepnik et al [51]	✓	✓	—	—	—	—	—
**Asthma (n=4)**								
	Chan et al [52]	✓	✓	✓	—	✓	—	—
	Cook et al [53]	—	—	—	✓	—	—	—
	Fedele et al [54]	—	✓	✓	✓	—	—	—
	Kosse et al [55]	✓	—	—	—	✓	—	—
**Cardiovascular disease (n=3)**								
	Agboola et al [56]	✓	—	—	✓	—	✓	✓
	Goyal et al [57]	✓	✓	✓	✓	✓	—	—
	Sakakibara et al [58]	✓	—	✓	—	—	—	—
**Type 1 diabetes (n=3)**								
	Goyal et al [59]	—	✓	✓	—	—	—	—
	Ryan et al [60]	✓	✓	✓	—	—	✓	—
	Sieber et al [61]	✓	—	—	—	—	—	—
**Type 2 diabetes (n=3)**								
	Desveaux et al [62]	—	✓	✓	✓	—	✓	—
	Goh et al [63]	—	✓	—	—	—	—	—
	Kleinman et al [64]	✓	—	—	✓	—	—	—
**Other (n=11)**								
	Bot et al [65]	✓	✓	✓	—	✓	—	—
	Hardinge et al [66]	✓	✓	✓	—	—	✓	—
	Isetta et al [67]	—	✓	—	—	—	—	—
	Kaplan et al [68]	✓	✓	✓	—	✓	✓	—
	Langius**-**Eklof et al [69]	✓	—	—	—	✓	—	—
	Ong et al [70]	✓	—	✓	✓	—	—	—
	Pham et al [71]	✓	✓	✓	✓	—	—	✓
	Serrano et al [34]	✓	✓	✓	—	—	—	✓
	Taki et al [72]	✓	✓	—	✓	—	✓	✓
	Thies et al [73]	✓	✓	✓	—	—	✓	—
	Toro**-**Ramos et al [74]	✓	✓	—	—	✓	—	—

^a^Analytic indicators of engagement used in reviewed studies.

^b^Not applicable.

#### Number of Measures

Of the analytic indicators identified in this review, the number of measures recorded by users on an app was the most commonly used indicator of engagement with mHealth apps for chronic conditions. Researchers evaluated a range of measures that aligned with their target chronic condition, such as blood glucose [56,60,61,64,73], weight [56,73,74], symptoms [66,68,69], patient-reported outcomes [38,46,52,65,71], diary entries [47,66], and steps [51]. There was some overlap in the types of measures being collected across apps targeting the same chronic conditions, such as the number of blood glucose readings recorded as an indicator of engagement with diabetes apps. Overall, the target chronic condition and functionality of the app under study ultimately determined which measures would be collected and subsequently reported as an analytic indicator of engagement.

#### Frequency of Interactions

The frequency of interactions logged was the second most prevalent analytic indicator of engagement. Researchers often chose to complement assessing the number of measures recorded on an app with the frequency by which the measures were recorded. Stratifying frequency of interactions by specific date ranges was also common; Davies et al measured the number of users who used a mental health app at least once after 1 week, 4 weeks, and 20 weeks [38]. They also applied within-date range indicators such as the number of users who used the app once, 2 to 3 times, 4 to 6 times, or 6 or more times per week. Some researchers assigned a benchmark number of days to signify engagement, such as Isetta et al who measured the number of users who engaged with an app for sleep apnea on at least 66% of all days in the study [67]. Others assigned significance to a specific day and considered reaching it as an indicator of engagement, such as Jamison et al who measured the number of users who continued to submit daily assessments of their chronic pain after 90 and 180 days [48]. Layering this analytic indicator over other indicators added temporal context to better understand how users were engaging over time.

#### Number of Features

The range of features accessed by users in an app was frequently measured as an analytic indicator of engagement. Researchers primarily logged (1) the number of features accessed and (2) the number of times each feature was accessed. In their trial of the Veterans Affairs' Comprehensive Assistance for Family Caregivers Program where users were provided with access to a suite of 6 apps for posttraumatic stress disorder (PTSD) self-management, Frisbee et al measured the number of unique apps used in the suite [39]. To better understand user preferences between 2 features of their app for schizophrenia self- management, Ben-Zeev et al measured the number of times users chose the video feature over the written content feature [36]. Our research group proposed exploring whether users would access all the features made available in their app for prostate cancer survivorship care, particularly whether users would enable caregiver permissions or write notes to document changes in their care [71]. Overall, researchers applied this analytic indicator to explore the breadth of app engagement and inform feature popularity and relevance for the target population.

#### Number of Log-Ins

The number of log-ins or sessions logged by users continues to be a commonly used analytic indicator of engagement. This indicator was often coupled with the frequency of interactions logged to standardize counts. Researchers also frequently measured the number of users who opened an app at least once to segment them from users who had downloaded the app but never logged any subsequent activity. Owen et al made both these associations by measuring the number of sessions logged by users on their PTSD self-management app, as well as the number of users who logged at least one session on the first day, week, and month post download [42]. Researchers used this analytic indicator to reflect the shift from adoption to habituation, with a greater number of log-ins or sessions denoting greater engagement.

**Table 2:** Descriptive overview of app structures across study characteristics and analytic indicators.

Characteristics		Structured (N=7), n (%)	Hybrid (N=24), n (%)	Unstructured (N=10), n (%)
**Chronic condition**				
	Mental health (n=12)	2 (29)	6 (25)	4 (40)
	Chronic pain (n=5)	2 (29)	3 (13)	0 (0)
	Asthma (n=4)	1 (14)	3 (13)	0 (0)
	Cardiovascular disease (n=3)	0 (0)	2 (8)	1 (10)
	Type 1 diabetes (n=3)	1 (14)	1 (4)	1 (10)
	Type 2 diabetes (n=3)	0 (0)	1 (4)	2 (20)
	Other (n=11)	1 (14)	8 (33)	2 (20)
**Segmentation**				
	Yes (n=19)	1 (14)	12 (50)	6 (60)
	No (n=14)	4 (47)	7 (29)	3 (30)
**Analytic indicators**				
	Number of measures (n=31)^a^	6 (86)	21 (88)	4 (40)
	Frequency of interactions (n=30)	4 (57)	18 (75)	8 (80)
	Number of features (n=20)^a^	2 (29)	10 (42)	8 (80)
	Number of log-ins (n=19)	4 (57)	12 (50)	3 (30)
	Number of modules (n=12)	2 (29)	10 (42)	0 (0)
	Time spent (n=11)	0 (0)	8 (33)	3 (30)
	Number of pages (n=7)	0 (0)	4 (17)	3 (30)
**Application of engagement data**				
	Descriptive (n=24)	7 (100)	13 (54)	4 (40)
	Inferential (n=17)	0 (0)	11 (46)	6 (60)
**Study design**				
	Experimental (n=21)	3 (43)	13 (54)	5 (50)
	Quasi-experimental (n=7)	0 (0)	6 (25)	1 (10)
	Observational (n=13)	4 (57)	5 (21)	4 (40)
**Number of indicators**				
	1 (n=5)	1 (14)	3 (13)	1 (1)
	2 (n=10)	2 (29)	4 (17)	4 (40)
	3 (n=8)	3 (43)	4 (17)	1 (10)
	4 (n=10)	1 (14)	6 (25)	3 (30)
	5 (n=7)	0 (0)	6 (25)	1 (10)
	6 (n=1)	0 (0)	1 (4)	0 (0)

^a^*P*<.05.

#### Number of Modules

When defining analytic indicators for categorization, we differentiated between unrestricted and restricted data collection. Unrestricted data collection was defined as data that could be entered into an app at a frequency or volume dictated by the user, such as the number of blood glucose readings or medications recorded [64]. Restricted data collection was defined as requiring the user to enter data according to a set frequency or volume, such as a list of assigned articles to be read [74] or challenges to be completed [57]. We coded studies reporting unrestricted data collection as *number of measures* and coded studies reporting restricted data collection as *number of modules*. A range of studies measured the number of outcome surveys completed from those assigned [45,68,75]. Others assessed the number of videos watched from a playlist [36,55], educational modules completed [52], or self-care advice accessed [69]. Overall, researchers studying apps with modular content considered module completion to be indicative of engagement and consequently, tracked module progression and completion rates.

#### Time Spent

The amount of time that users engaged with an app was considered by a subset of researchers to be an analytic indicator of engagement. Researchers measured the time spent on unique sections of an app [66], the time spent on unique pages [56], the length of a unique session [38,42,43,71], the length between unique sessions [72], and the total time spent on an app [62,68,73]. Davies et al also segmented sessions by those that were in the 30- to 60-second range [38]. Measuring time spent engaging with an app helped researchers to distinguish between exploratory and purposeful engagement; a rapid succession of short page views was indicative of scanning through content, whereas prolonged viewing suggested greater intention and interest in content. Overall, this analytic indicator informed defining accurate session duration parameters to track session-based analytics.

#### Number of Pages

The number of pages accessed by users was logged by researchers to reflect overall patterns of app engagement and discoverability of specific content. Kuhn et al measured the number and content of pages visited by users in their app for PTSD self-management, as did other researchers [38,41,71]. Taki et al combined session analytics with page analytics and measured the number of pages viewed per session in their app for obesity self-management [72]. Owen et al recorded click stream data documenting their users’ navigation through page content [42]. Insights gleaned from this analytic indicator provided researchers with a broader understanding of the user journey through an app and drew attention to specific content that might drive engagement.

#### Conceptual Categories of Analytic Indicators

We sought to conceptually clarify the 7 identified analytic indicators by grouping them according to the 4 categories that constitute the quantitative conceptualization of engagement: amount, duration, breadth, and depth [11,12]. Table 3 presents an overview of the categories, their comprised analytic indicators, and the number of reviewed studies that fall into each category. The focus of most reviewed studies was on the depth (76%, 31/41) and amount of engagement (73%, 30/41). There was less attention on the breadth (49%, 20/41) and duration (27%, 11/41) of engagement. TThese findings suggest that a subset of researchers are either not measuring the breadth and duration of engagement in their mHealth evaluations or underreporting the findings.

### Application of Engagement Data

Of the 41 studies included for review, 24 presented, described, or summarized the data generated from applying analytic indicators to measure engagement. The remaining 17 studies used or planned to use these data to infer a relationship between engagement patterns and intended outcomes.

#### Clinical Outcomes

Over half of all researchers assessed the relationship between engagement and clinical outcomes (53%, 9/17). Toro-Ramos et al measured the number of weeks users engaged with their hypertension self-management app and found that users with sustained usage across 19 weeks experienced significant reductions in systolic blood pressure and weight [74]. In their trial of an app for PTSD self-management, Kuhn et al applied the number of days and weeks users engaged with the app as a predictor variable for changes in PTSD symptoms but did not find a significant relationship [41]. Goyal et al segmented all users who reported 5 or more blood glucose readings a day into a subgroup for secondary analyses and found a significant relationship between increased readings and improved glycated hemoglobin after 6 months [59]. They also identified a significant interaction between users who entered a reading on at least three days a week, and improved daily blood glucose self-monitoring. Overall, there was evidence of predictive validity across reviewed studies, with engagement correlating with improved clinical outcomes. However, the majority of analyses conducted to establish this predictive validity relied on nonexperimental variations in engagement due to nonadherence or implementation infidelity. Future evaluations assessing the relationship between engagement and clinical outcomes should consider alternative trial designs with multiple randomizations to ensure that findings are not biased by confounding [76-78].

**Table 3:** Conceptual categories of analytic indicators.

Category and analytic indicators		Studies, n (%)
**Amount**		
	Frequency of interactions	30 (73)
	Number of log-ins	30 (73)
Duration: Time spent		11 (27)
**Breadth**		
	Number of features	20 (49)
	Number of pages	20 (49)
**Depth**		
	Number of modules	31 (76)
	Number of measures	31 (76)

#### Engagement Outcomes

Many researchers sought to investigate the effect of engagement behaviors on other engagement outcomes (53%, 9/17). In their study examining engagement with a weight loss app, Serrano et al applied classification and regression tree methods to identify subgroups with unique engagement behaviors [79]. They were able to distinguish highly engaged subgroups by the number of customizations made to the diet and exercise features of the app. Ben-Zeev et al found that participants who engaged with their schizophrenia self-management app for a period of 5 to 6 months also had a higher frequency of interactions and engaged 4.3 days per week on average [37]. Torous et al also characterized engagement for a schizophrenia self-management app through fitting frequency of interaction data to a piecewise power law distribution [44]. They found that future use with the app is directly related to prior app use, suggesting that those who engage with the app more often will have a higher probability of app engagement in the future. In their trial of a caloric-monitoring app for type 2 diabetes self-management, Goh et al applied latent-class growth modeling to delineate 8-week trajectories of app engagement [63]. They were able to identify 3 distinct app trajectories based on the frequency of interactions and also associate patient characteristics with these trajectories. In summary, there were strong predictive relationships between numerous engagement domains. This finding motivates establishing complementary domains across multiple contexts to optimize data triangulation.

#### Utilization Outcomes

Two studies proposed to evaluate the impact of engagement patterns on health care utilization outcomes (12%, 2/17). Kaplan et al plan to examine the impact of sustained engagement over time with an app for pediatric cystic fibrosis and inflammatory bowel disease self-management on the number of hospitalizations and emergency department visits [68]. However, they anticipate that changes in these outcomes may not be realized in a 6-month intervention period. Our research group is evaluating a prostate cancer survivorship app [71] and aims to investigate the relationship between (1) the number of patient-reported outcome measures completed and (2) the frequency of interactions logged on the number of in-clinic visits for prostate cancer–related concerns. Altogether, the limited sample of reviewed studies suggests that the relationship between engagement and utilization outcomes is underdeveloped and warrants further study.

The Fisher exact test of independence indicated that studies of structured apps were more likely to only report descriptive statistics on engagement data (7/7, *P*=.04). In addition, most studies that applied inferential statistics also measured the frequency of interactions logged (16/17, *P*=.014). Most researchers who did not segment users into cohorts based on engagement data only reported descriptive statistics on their engagement data (13/14, *P*<.001), while researchers who segmented their users into cohorts were more likely to conduct subgroup analyses and infer properties of the larger clinical population (14/19, *P*<.001). Table 4 provides a descriptive overview of studies applying descriptive or inferential analyses on engagement data.

### Attrition Type and Analyses

The majority of reviewed studies did not report on attrition (70%, 23/33). Of the 10 studies that did, 5 reported on dropout attrition (50%), 4 reported on nonusage attrition (40%), and 1 reported on both phenomena (10%). Researchers were more likely to descriptively summarize raw attrition proportions than statistically analyze them (70%, 7/10). Those that conducted comparisons across attrition curves used Kaplan-Meier survival curves (10%, 1/10), Cox regression models (10%, 2/10), and latent class growth models (10%, 1/10).

**Table 4:** Descriptive overview of descriptive and inferential engagement data application across study characteristics and analytic indicators.

Characteristics		Descriptive (N=24), n (%)	Inferential (N=17), n (%)
**Chronic condition**			
	Mental health (n=12)	6 (25)	6 (35)
	Chronic pain (n=5)	4 (17)	1 (6)
	Asthma (n=4)	3 (13)	1 (6)
	Cardiovascular disease (n=3)	2 (8)	1 (6)
	Type 1 diabetes (n=3)	2 (8)	1 (6)
	Type 2 diabetes (n=3)	2 (8)	1 (6)
	Other (n=11)	5 (21)	6 (35)
**Segmentation**			
	Yes (n=19)^a^	5 (21)	14 (82)
	No (n=14)^a^	13 (54)	1 (6)
**Analytic indicators**			
	Number of measures (n=31)	20 (83)	11 (65)
	Frequency of interactions (n=30)^a^	14 (58)	16 (94)
	Number of features (n=20)	11 (46)	9 (53)
	Number of log-ins (n=19)	12 (50)	7 (41)
	Number of modules (n=12)	7 (29)	5 (29)
	Time spent (n=11)	8 (33)	3 (18)
	Number of pages (n=7)	3 (13)	4 (24)
**Structure**			
	Structured (n=7)^a^	7 (29)	0 (0)
	Hybrid (n=24)	13 (54)	11 (65)
	Unstructured (n=10)	4 (17)	6 (35)
**Study design**			
	Experimental (n=21)	13 (54)	8 (47)
	Quasi-experimental (n=7)	4 (17)	3 (18)
	Observational (n=13)	7 (29)	6 (35)
**Number of indicators**			
	1 (n=5)	3 (13)	2 (12)
	2 (n=10)	7 (29)	3 (18)
	3 (n=8)	3 (13)	5 (29)
	4 (n=10)	7 (29)	3 (18)
	5 (n=7)	3 (13)	4 (24)
	6 (n=1)	1 (4)	0 (0)

^a^*P*<.05.

## Discussion

### Principal Findings

In conducting this scoping review, we sought to catalog the range of analytic indicators being used in evaluations of consumer mHealth apps for chronic conditions. We applied Arksey and O’Malley’s methods of reporting and provided a descriptive analysis of the extent, nature, and distribution of analytic indicators across 41 studies, as well as a narrative and thematic summary of collected data [27]. The average mHealth evaluation included for review was a two-group pretest-posttest RCT of a hybrid-structured app for mental health self- management, had 103 participants, lasted 5 months, did not provide access to health care provider services, measured 3 analytic indicators of engagement, segmented users based on engagement data, applied engagement data for descriptive analyses, and did not report on attrition.

#### Analytic Indicators

Our results indicate that researchers are measuring engagement across 7 analytic indicators, specifically: (1) the number of measures recorded, (2) the frequency of interactions logged, (3) the number of features accessed, (4) the number of log-ins or sessions logged, (5) the number of modules or lessons started or completed, (6) time spent engaging with the app, and (7) the number or content of pages accessed. We found that the researchers favored evaluating the number of measures recorded on an app as an indicator of engagement, closely followed by the frequency of interactions logged. We also found that both these indicators were most often used to assess hybrid and unstructured apps; these 2 app structures also made up the majority of apps under review.

We noted that researchers were least likely to measure the number of pages accessed and time spent engaging with the app; the latter indicator was mostly reported descriptively (73%, 8/11). This finding was surprising given the historical popularity of these indicators for measuring engagement with Web-based interventions [17,23,80]. The breadth and duration categories that conceptually comprise these analytic indicators were also deprioritized. We propose that these indicators are falling out of favor because of the growing recognition that users engage differently with apps. Users perceive apps to be a short-term commitment [81] and access app-based content sporadically for shorter periods of time compared with Web-based interventions [82]. Recent research by Morrison et al comparing patterns of engagement with a stress management intervention delivered via website versus app mitigated these differences by significantly reducing the number of pages on the app version of the intervention compared with the website [83]. They subsequently found that app users logged in twice as often but spent half as much time engaging compared with website users. They did not report the number of pages accessed or time spent engaging with the app as indicators of engagement. This body of research, in conjunction with our own findings, suggests that researchers evaluating mHealth apps for self-managing chronic conditions should refrain from measuring and reporting these 2 analytic indicators of engagement unless they are expressly relevant to the app under study.

Our identification of the number of measures recorded on an app as an analytic indicator of engagement deviates from previous research by Sieverink et al on usage and adherence to eHealth interventions [17], which found no evidence that researchers were operationalizing constructs in this way. Our focus on reviewing studies of mHealth apps for self-managing chronic conditions may explain this finding, as these interventions encourage users to systematically record data and capture the variability of their disease state over time [84]. In thinking of the frequency of interactions logged as a common analytic indicator of engagement, we note that there has been a shift toward on-demand apps with features and functionality that users can engage with at their own discretion. Benchmarking engagement by time range provides more context on a user’s intentions and needs than just the total amount of engagement.

We did not observe any significant differences between the number or type of analytic indicators used to measure engagement across chronic conditions. Researchers applied indicators that were relevant to the features and functionality of their app. For example, studies of apps for diabetes self-management often measured the number of blood glucose readings due to the popularity of this feature but never measured the number of modules or lessons because these features were not offered to users. In a recent review on the barriers and facilitators of engagement with remote measurement technology for managing health, Simblett et al found that studies were reporting idiosyncratic measures of engagement and adherence that were not comparable across studies [26]. Their findings align with our own, and support Yardley et al’s assertion that effective engagement is defined in relation to the purpose of a specific intervention and can only be established empirically in the context of that intervention [14]. Although Simblett et al call for less variation in how engagement is quantitatively measured across studies, we propose that researchers continue to apply context-specific analytic indicators but report them more systematically to enable cross-study comparison. Researchers might consider categorizing indicators according to the 7 domains identified in this research and providing detailed specifications on the analytic tags required to implement each indicator. When reporting on indicators, researchers should specify that they are measuring the construct of engagement and then catalog each domain. This practice may contribute to greater taxonomic consensus by curbing the arbitrary reporting of engagement-related constructs identified in this review.

#### Application of Engagement Data

Although researchers measured, on average, 3 indicators in a single study, the majority reported findings descriptively and did not further investigate how engagement with an app contributed to its impact on health and well-being. This finding suggests that researchers are gaining nuanced insights into how users are engaging with their apps but are not conducting inferential analyses to characterize effective engagement for improved outcomes. Relating analytic engagement patterns to behavior change and intended outcomes has been advocated across the behavioral and computational sciences [14,15,24,85,86], with recent efforts made to equip researchers with strategies for performing inferential analyses on engagement data [22,87,88]. Our analyses indicated that studies of structured apps were more likely to only report descriptive statistics on engagement data. Given that structured apps primarily require users to follow a predetermined engagement pathway and complete a series of milestones, it is reasonable for researchers to report on completion rates and identify drop-off points. However, it may be helpful to conduct inferential analyses to understand if completion of an app-mediated program is required to achieve intended outcomes, or whether users may derive proportional benefits from progressing through stages of the program. Of the studies that applied inferential statistics, most measured the number of days, week, or months users engaged with an app. This finding suggests that researchers consider a temporal understanding of engagement to be important in determining a predictive effect on intended outcomes.

**Figure 2:**
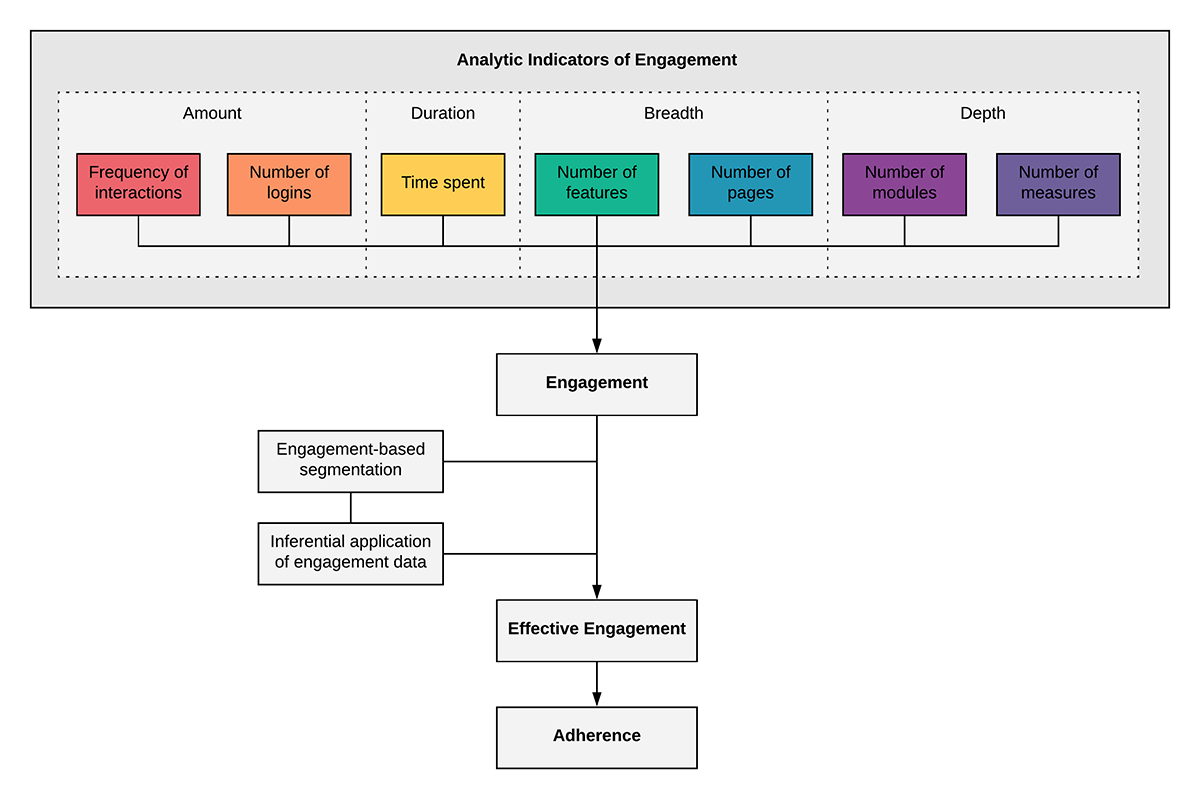
Process model of methodological continuum for evaluating mobile health engagement to adherence.

#### Recommendations

In their systematic review, Sieverink et al found that over half of all reviewed studies measured adherence to eHealth interventions using a single analytic indicator, and a quarter used 2 indicators [17]. The authors conclude that a limited but deliberate set of only one of 2 different indicators in accordance with the goal of the technology is sufficient to operationalize adherence. On reviewing how researchers were operationalizing adherence, they found that the majority reported adherence only in terms of how an intervention was used. The absence of a comparison to a threshold for intended use renders this operationalization incongruent with the definition of adherence. Instead, we propose that it aligns with the current understanding of engagement, which is more exploratory in nature and thus supports applying a greater number of analytic indicators.

In contrast to Sieverink et al’s findings, the majority of our reviewed studies applied between 2 and 4 analytic indicators to measure engagement. This variance suggests that researchers are starting to recognize a conceptual and methodological distinction between the constructs of engagement and adherence. From these findings, we make the following recommendation: researchers seeking to gain a preliminary understanding of how users are engaging with their app are encouraged to apply all relevant analytic indicators from those identified in this review. Multimedia Appendix 2 presents data that may support researchers to select indicators that have previously been measured for their target chronic condition or for an app with similar features and functionality. Upon generation of analytic findings, researchers might consider segmenting users by engagement behaviors to interrogate the data and refine their engagement models. Conducting inferential subgroup analyses with engagement as a predictor of observed health outcomes might uncover potential patterns of effective engagement and inform an operationalization of intended use. In this way, measuring engagement can be positioned on a methodological continuum toward determining adherence. Figure 2 presents a process model of our recommendations.

During our full-text review, we excluded a large number of studies because they did not include objective, quantifiable measurements using log data analytics. Some studies had users self-report their engagement, whereas others omitted reporting engagement altogether and solely related findings on app efficacy. One possible explanation for this gap might be that researchers are unfamiliar with how to derive analytic insights from their app. From our experience, the process of tagging interaction data to enable analytic insights requires deliberate foresight. A shared understanding between a researcher and a software developer of the research questions being answered is critical to determine how analytics data should be modeled. Multimedia Appendix 3 presents a use case for applying analytic tags to evaluate effective engagement.

Our final recommendation concerns the reporting of attrition in data-driven mHealth evaluations. In 2005, Eysenbach published landmark work on the *law of attrition* [10], which was his observation that a substantial portion of participants in eHealth trials stop using the intervention before study end. He posits that attrition is a fundamental characteristic and methodological challenge in the evaluation of eHealth interventions and recommends that “usage metrics and determinants of attrition should be highlighted, measured, analyzed, and discussed” [10]. Our findings suggest that this counsel has not fully translated into practice in the mHealth field. There is less inclination to log and report on analytic indicators of disengagement. We encourage researchers to attribute the same value to attrition data as they currently do to engagement data, as both constructs provide consequential insights into the viability of an app in the real world.

### Limitations

Some methodological limitations of our scoping review warrant discussion, the most significant being that we only reviewed articles published over a 2-year period. This sampling frame may not have captured a representative sample of mHealth literature. As such, we may have missed relevant studies published before November 2015 and after November 2017 that would have met our eligibility criteria. While we acknowledge that our sampling frame is limited in scoping the entire field of mHealth, we believe it captures the application of analytics within the field of mHealth. From our review of the literature before conducting our search, we identified a paucity of papers that focused on mHealth log data analyses. The systematic review on usage-based adherence to eHealth interventions conducted by Sieverink et al reviewed 62 papers, of which 7 were on smartphone-based interventions [17]. Of those 7 papers, 5 were published after 2016, and the other 2 were both published in 2013. Perski et al conducted a systematic review on engagement with digital behavior change interventions that comprised all studies up to November 2015 [11]. They reviewed 113 studies, of which 13 were on mobile phone–based interventions. Only 4 of those studies applied log data analyses to study engagement with the intervention. These insights confirm that our scoping review did not include all studies that applied log data analyses to study engagement with mHealth apps. However, they also suggest that the number of studies we omitted is small. Our sampling frame of November 2015 to November 2017 directly follows Perski et al’s review and includes 41 studies to address our specific research questions. For these reasons, we posit that our sample is sufficiently robust to provide a representative understanding of how analytics are being applied to study engagement with mHealth apps. Due to limited resources, only 1 reviewer conducted the electronic searches and screened all titles and abstracts against eligibility criteria, thereby potentially introducing bias. We did not assess the quality of included articles; however, this is in line with our review framework, which does not mandate this methodological practice. Finally, we did not map analytic indicators to the 14 identified engagement-related constructs for analysis. We acknowledge that conceptual differences exist between some of these constructs (eg, usage, feasibility, and adherence), and it is possible to use multiple constructs in the same study. However, we reviewed each construct and its analytic operationalizations separately during our data extraction process and could not discern significant differences. As such, we feel that we have included a homogenous body of research in this review and provided accurate insights into how researchers have used analytic indicators to measure engagement.

### Conclusions

To date, the potential for mHealth apps to positively impact chronic health outcomes has not yet been realized [89]. This is, in part, due to the difficulties of generating a solid evidence base to guide clinical, policy, and regulatory decision making [90]. Indeed, the mHealth field has been reproached for arguing that apps warrant *digital exceptionalism* given the iterative nature of their design and the prohibitive cost of trials compared with their perceived level of risk [91]. We propose that our review supports researchers to harness these natural attributes for conducting data-driven evaluations of digitally mediated behavior change. Without objective knowledge of how users engage with an app to care for themselves, the mechanisms of action that underlie complex models of digitally mediated behavior change cannot be identified.

Our proposed library of analytic indicators to evaluate effective engagement with consumer mHealth apps for chronic conditions may be of value to researchers as a resource to support their evaluative practice. Researchers can systematically incorporate these analytic indicators into their study measures by adding analytic tags to their app’s source code, allowing them to measure engagement without creating user burden or reactivity. Once generated, these data can be used in inferential analyses to delineate relationships with observed health outcomes. Researchers can further interrogate these data by conducting rapid cycles of research and development to validate hypothesized models of effective engagement. On the basis of these insights, researchers can (1) build a cumulative body of evidence for how users should engage with their app to achieve intended outcomes, (2) incrementally improve their app to optimize effective engagement, and (3) determine the optimal digital dose of effective engagement with their app for validation in a definitive trial to meet required levels of evidence for procurement and distribution [92]. Successful implementation of these practices may elevate the discourse of these apps beyond the coarse evaluations and monolithic policy recommendations against their value in health care.

Raising the standard of mHealth app efficacy through measuring analytic indicators of engagement may enable greater confidence in the causal impact of apps on improved chronic health and well-being. It is this opportunity afforded by data-driven research to close the gap between promised and realized health benefits that is most meaningful.

## Appendix

Multimedia Appendix 1 Scoping review search strategy.

Multimedia Appendix 2 Full dataset of coded analytic indicators.

Multimedia Appendix 3 Practical considerations for applying analytic indicators to evaluate effective engagement.

## Abbreviations

eHealth: electronic health
IQR: interquartile range
mHealth: mobile health
PTSD: posttraumatic stress disorder
RCT: randomized controlled trial

## References

[1] Byambasuren O, Sanders S, Beller E, Glasziou P (2018). Prescribable mHealth apps identified from an overview of systematic reviews. NPJ Digit Med.

[2] Proudfoot J, Clarke J, Birch MR, Whitton AE, Parker G, Manicavasagar V, Harrison V, Christensen H, Hadzi-Pavlovic D (2013). Impact of a mobile phone and web program on symptom and functional outcomes for people with mild-to-moderate depression, anxiety and stress: a randomised controlled trial. BMC Psychiatry.

[3] Clarke J, Proudfoot J, Birch MR, Whitton AE, Parker G, Manicavasagar V, Harrison V, Christensen H, Hadzi-Pavlovic D (2014). Effects of mental health self-efficacy on outcomes of a mobile phone and web intervention for mild-to-moderate depression, anxiety and stress: secondary analysis of a randomised controlled trial. BMC Psychiatry.

[4] Ivanova E, Lindner P, Ly KH, Dahlin M, Vernmark K, Andersson G, Carlbring P (2016). Guided and unguided acceptance and commitment therapy for social anxiety disorder and/or panic disorder provided via the internet and a smartphone application: a randomized controlled trial. J Anxiety Disord.

[5] Roepke AM, Jaffee SR, Riffle OM, McGonigal J, Broome R, Maxwell B (2015). Randomized controlled trial of SuperBetter, a smartphone-based/internet-based self-help tool to reduce depressive symptoms. Games Health J.

[6] Laing BY, Mangione CM, Tseng CH, Leng M, Vaisberg E, Mahida M, Bholat M, Glazier E, Morisky DE, Bell DS (2014). Effectiveness of a smartphone application for weight loss compared with usual care in overweight primary care patients: a randomized, controlled trial. Ann Intern Med.

[7] Direito A, Jiang Y, Whittaker R, Maddison R (2015). Smartphone apps to improve fitness and increase physical activity among young people: protocol of the Apps for IMproving FITness (AIMFIT) randomized controlled trial. BMC Public Health.

[8] Turner-McGrievy G, Tate D (2011). Tweets, apps, and pods: results of the 6-month Mobile Pounds Off Digitally (Mobile POD) randomized weight-loss intervention among adults. J Med Internet Res.

[9] Holmen H, Torbjørnsen A, Wahl AK, Jenum AK, Småstuen MC, Arsand E, Ribu L (2014). A mobile health intervention for self-management and lifestyle change for persons with type 2 diabetes, part 2: one-year results from the Norwegian randomized controlled trial RENEWING HEALTH. JMIR Mhealth Uhealth.

[10] Eysenbach G (2005). The law of attrition. J Med Internet Res.

[11] Perski O, Blandford A, West R, Michie S (2017). Conceptualising engagement with digital behaviour change interventions: a systematic review using principles from critical interpretive synthesis. Transl Behav Med.

[12] O'Brien Hl, Toms EG (2008). What is user engagement? A conceptual framework for defining user engagement with technology. J Am Soc Inf Sci.

[13] Pham Quynh, Graham Gary, Lalloo Chitra, Morita Plinio P, Seto Emily, Stinson Jennifer N, Cafazzo Joseph A (2018). An Analytics Platform to Evaluate Effective Engagement With Pediatric Mobile Health Apps: Design, Development, and Formative Evaluation. JMIR Mhealth Uhealth.

[14] Yardley L, Spring BJ, Riper H, Morrison LG, Crane DH, Curtis K, Merchant GC, Naughton F, Blandford A (2016). Understanding and promoting effective engagement with digital behavior change interventions. Am J Prev Med.

[15] Michie S, Yardley L, West R, Patrick K, Greaves F (2017). Developing and evaluating digital interventions to promote behavior change in health and health care: recommendations resulting from an international workshop. J Med Internet Res.

[16] Barello S, Triberti S, Graffigna G, Libreri C, Serino S, Hibbard J, Riva G (2016). eHealth for patient engagement: a systematic review. Front Psychol.

[17] Sieverink F, Kelders SM, van Gemert-Pijnen JE (2017). Clarifying the concept of adherence to eHealth technology: systematic review on when usage becomes adherence. J Med Internet Res.

[18] Hervatis V, Loe A, Barman L, O'Donoghue J, Zary N (2015). A conceptual analytics model for an outcome-driven quality management framework as part of professional healthcare education. JMIR Med Educ.

[19] Kotz D, Lord SE, O'Malley AJ, Stark L, Marsch LA (2018). Workshop on emerging technology and data analytics for behavioral health. JMIR Res Protoc.

[20] Van Gemert-Pijnen JE, Kelders SM, Bohlmeijer ET (2014). Understanding the usage of content in a mental health intervention for depression: an analysis of log data. J Med Internet Res.

[21] Sieverink F, Kelders SM, Braakman-Jansen LM, van Gemert-Pijnen JE (2014). The added value of log file analyses of the use of a personal health record for patients with type 2 diabetes mellitus: preliminary results. J Diabetes Sci Technol.

[22] Sieverink F, Kelders S, Poel M, van Gemert-Pijnen L (2017). Opening the black box of electronic health: collecting, analyzing, and interpreting log data. JMIR Res Protoc.

[23] Arden-Close EJ, Smith E, Bradbury K, Morrison L, Dennison L, Michaelides D, Yardley L (2015). A visualization tool to analyse usage of web-based interventions: the example of Positive Online Weight Reduction (POWeR). JMIR Hum Factors.

[24] Hekler EB, Klasnja P, Riley WT, Buman MP, Huberty J, Rivera DE, Martin CA (2016). Agile science: creating useful products for behavior change in the real world. Transl Behav Med.

[25] Short C, DeSmet A, Woods C, Williams SL, Maher C, Middelweerd A, Müller AM, Wark PA, Vandelanotte C, Poppe L, Hingle MD, Crutzen R (2018). Measuring engagement in eHealth and mHealth behavior change interventions: viewpoint of methodologies. J Med Internet Res.

[26] Simblett S, Greer B, Matcham F, Curtis H, Polhemus A, Ferrão J, Gamble P, Wykes T (2018). Barriers to and facilitators of engagement with remote measurement technology for managing health: systematic review and content analysis of findings. J Med Internet Res.

[27] Arksey H, O'Malley L (2005). Scoping studies: towards a methodological framework. Int J Soc Res Methodol.

[28] Levac D, Colquhoun H, O'Brien KK (2010). Scoping studies: advancing the methodology. Implement Sci.

[29] (2010). Global Status Report on Noncommunicable Diseases. World Health Organization.

[30] Elsevier.

[31] Kelders SM, Kok RN, Ossebaard HC, Van Gemert-Pijnen JE (2012). Persuasive system design does matter: a systematic review of adherence to web-based interventions. J Med Internet Res.

[32] IBM Corporation.

[33] Moher D, Liberati A, Tetzlaff J, Altman DG, PRISMA Group (2009). Preferred reporting items for systematic reviews and meta-analyses: the PRISMA statement. PLoS Med.

[34] Serrano KJ, Coa KI, Yu M, Wolff-Hughes DL, Atienza AA (2017). Characterizing user engagement with health app data: a data mining approach. Transl Behav Med Internet.

[35] Beiwinkel T, Kindermann S, Maier A, Kerl C, Moock J, Barbian G, Rössler W (2016). Using smartphones to monitor bipolar disorder a symptoms: a pilot study. JMIR Ment Health.

[36] Ben-Zeev D, Brian R, Aschbrenner KA, Jonathan G, Steingard S (2018). Video-based mobile health interventions for people with schizophrenia: bringing the "pocket therapist" to life. Psychiatr Rehabil J.

[37] Ben-Zeev D, Scherer EA, Gottlieb JD, Rotondi AJ, Brunette MF, Achtyes ED, Mueser KT, Gingerich S, Brenner CJ, Begale M, Mohr DC, Schooler N, Marcy P, Robinson DG, Kane JM (2016). mHealth for schizophrenia: patient engagement with a mobile phone intervention following hospital discharge. JMIR Ment Health.

[38] Davies EB, Craven MP, Martin JL, Simons L (2017). Proportionate methods for evaluating a simple digital mental health tool. Evid Based Ment Health.

[39] Frisbee KL (2016). Variations in the use of mHealth tools: the VA mobile health Study. JMIR Mhealth Uhealth.

[40] Kinderman P, Hagan P, King S, Bowman J, Chahal J, Gan L, McKnight R, Waldon C, Smith M, Gilbertson J, Tai S (2016). The feasibility and effectiveness of Catch It, an innovative CBT smartphone app. BJPsych Open.

[41] Kuhn E, Kanuri N, Hoffman JE, Garvert DW, Ruzek JI, Taylor CB (2017). A randomized controlled trial of a smartphone app for posttraumatic stress disorder symptoms. J Consult Clin Psychol.

[42] Owen JE, Jaworski BK, Kuhn E, Makin-Byrd KN, Ramsey KM, Hoffman JE (2015). mHealth in the wild: using novel data to examine the reach, use, and impact of PTSD coach. JMIR Ment Health.

[43] Pham Q, Khatib Y, Stansfeld S, Fox S, Green T (2016). Feasibility and efficacy of an mHealth game for managing anxiety: "Flowy" randomized controlled pilot trial and design evaluation. Games Health J.

[44] Torous J, Staples P, Slaters L, Adams J, Sandoval L, Onnela JP, Keshavan M (2017). Characterizing smartphone engagement for schizophrenia: results of a naturalist mobile health study. Clin Schizophr Relat Psychoses.

[45] Vansimaeys C, Zuber M, Pitrat B, Join-Lambert C, Tamazyan R, Farhat W, Bungener C (2017). Combining standard conventional measures and ecological momentary assessment of depression, anxiety and coping using smartphone application in minor stroke population: a longitudinal study protocol. Front Psychol.

[46] Wahle F, Kowatsch T, Fleisch E, Rufer M, Weidt S (2016). Mobile sensing and support for people with depression: a pilot trial in the wild. JMIR Mhealth Uhealth.

[47] Fortier MA, Chung WW, Martinez A, Gago-Masague S, Sender L (2016). Pain buddy: a novel use of m-health in the management of children's cancer pain. Comput Biol Med.

[48] Jamison RN, Jurcik DC, Edwards RR, Huang CC, Ross EL (2017). A pilot comparison of a smartphone app with or without 2-way messaging among chronic pain patients: who benefits from a pain app?. Clin J Pain.

[49] Jibb LA, Stevens BJ, Nathan PC, Seto E, Cafazzo JA, Johnston DL, Hum V, Stinson JN (2017). Implementation and preliminary effectiveness of a real-time pain management smartphone app for adolescents with cancer: a multicenter pilot clinical study. Pediatr Blood Cancer.

[50] Reade S, Spencer K, Sergeant JC, Sperrin M, Schultz DM, Ainsworth J, Lakshminarayana R, Hellman B, James B, McBeth J, Sanders C, Dixon WG (2017). Cloudy with a chance of pain: engagement and subsequent attrition of daily data entry in a smartphone pilot study tracking weather, disease severity, and physical activity in patients with rheumatoid arthritis. JMIR Mhealth Uhealth.

[51] Skrepnik N, Spitzer A, Altman R, Hoekstra J, Stewart J, Toselli R (2017). Assessing the impact of a novel smartphone application compared with standard follow-up on mobility of patients with knee osteoarthritis following treatment with hylan G-F 20: a randomized controlled trial. JMIR Mhealth Uhealth.

[52] Chan YY, Wang P, Rogers L, Tignor N, Zweig M, Hershman SG, Genes N, Scott ER, Krock E, Badgeley M, Edgar R, Violante S, Wright R, Powell CA, Dudley JT, Schadt EE (2017). The asthma mobile health study, a large-scale clinical observational study using ResearchKit. Nat Biotechnol.

[53] Cook KA, Modena BD, Simon RA (2016). Improvement in asthma control using a minimally burdensome and proactive smartphone application. J Allergy Clin Immunol Pract.

[54] Fedele DA, McConville A, Graham Thomas J, McQuaid EL, Janicke DM, Turner EM, Moon J, Abu-Hasan M (2018). Applying interactive mobile health to asthma care in teens (AIM2ACT): development and design of a randomized controlled trial. Contemp Clin Trials.

[55] Kosse RC, Bouvy ML, de Vries TW, Kaptein AA, Geers HC, van Dijk L, Koster ES (2017). mHealth intervention to support asthma self-management in adolescents: the ADAPT study. Patient Prefer Adherence.

[56] Agboola S, Palacholla RS, Centi A, Kvedar J, Jethwani K (2016). A multimodal mHealth intervention (FeatForward) to improve physical activity behavior in patients with high cardiometabolic risk factors: rationale and protocol for a randomized controlled trial. JMIR Res Protoc.

[57] Goyal S, Morita PP, Picton P, Seto E, Zbib A, Cafazzo JA (2016). Uptake of a consumer-focused mHealth application for the assessment and prevention of heart disease: the <30 days study. JMIR Mhealth Uhealth.

[58] Sakakibara BM, Ross E, Arthur G, Brown-Ganzert L, Petrin S, Sedlak T, Lear SA (2017). Using mobile-health to connect women with cardiovascular disease and improve self-management. Telemed J E Health.

[59] Goyal S, Nunn CA, Rotondi M, Couperthwaite AB, Reiser S, Simone A, Katzman DK, Cafazzo JA, Palmert MR (2017). A mobile app for the self-management of type 1 diabetes among adolescents: a randomized controlled trial. JMIR Mhealth Uhealth.

[60] Ryan EA, Holland J, Stroulia E, Bazelli B, Babwik SA, Li H, Senior P, Greiner R (2017). Improved A1C levels in type 1 diabetes with smartphone App use. Can J Diabetes.

[61] Sieber J, Flacke F, Link M, Haug C, Freckmann G (2017). Improved glycemic control in a patient group performing 7-point profile self-monitoring of blood glucose and intensive data documentation: an open-label, multicenter, observational study. Diabetes Ther.

[62] Desveaux L, Agarwal P, Shaw J, Hensel JM, Mukerji G, Onabajo N, Marani H, Jamieson T, Bhattacharyya O, Martin D, Mamdani M, Jeffs L, Wodchis WP, Ivers NM, Bhatia RS (2016). A randomized wait-list control trial to evaluate the impact of a mobile application to improve self-management of individuals with type 2 diabetes: a study protocol. BMC Med Inform Decis Mak.

[63] Goh G, Tan NC, Malhotra R, Padmanabhan U, Barbier S, Allen JC, Østbye T (2015). Short-term trajectories of use of a caloric-monitoring mobile phone app among patients with type 2 diabetes mellitus in a primary care setting. J Med Internet Res.

[64] Kleinman NJ, Shah A, Shah S, Phatak S, Viswanathan V (2017). Improved medication adherence and frequency of blood glucose self-testing using an m-Health platform versus usual care in a multisite randomized clinical trial among people with type 2 diabetes in India. Telemed J E Health.

[65] Bot BM, Suver C, Neto EC, Kellen M, Klein A, Bare C, Doerr M, Pratap A, Wilbanks J, Dorsey ER, Friend SH, Trister AD (2016). The mPower study, Parkinson disease mobile data collected using ResearchKit. Sci Data.

[66] Hardinge M, Rutter H, Velardo C, Shah SA, Williams V, Tarassenko L, Farmer A (2015). Using a mobile health application to support self-management in chronic obstructive pulmonary disease: a six-month cohort study. BMC Med Inform Decis Mak.

[67] Isetta V, Torres M, González K, Ruiz C, Dalmases M, Embid C, Navajas D, Farré R, Montserrat JM (2017). A new mHealth application to support treatment of sleep apnoea patients. J Telemed Telecare.

[68] Kaplan HC, Thakkar SN, Burns L, Chini B, Dykes DM, McPhail GL, Moore E, Saeed SA, Eslick I, Margolis PA, Opipari-Arrigan L (2017). Protocol of a pilot study of technology-enabled coproduction in pediatric chronic illness care. JMIR Res Protoc.

[69] Langius-Eklöf A, Crafoord MT, Christiansen M, Fjell M, Sundberg K (2017). Effects of an interactive mHealth innovation for early detection of patient-reported symptom distress with focus on participatory care: protocol for a study based on prospective, randomised, controlled trials in patients with prostate and breast cancer. BMC Cancer.

[70] Ong S, Jassal SV, Miller JA, Porter EC, Cafazzo JA, Seto E, Thorpe KE, Logan AG (2016). Integrating a smartphone-based self-management system into usual care of advanced CKD. Clin J Am Soc Nephrol.

[71] Pham Q, Cafazzo JA, Feifer A (2017). Adoption, acceptability, and effectiveness of a mobile health app for personalized prostate cancer survivorship care: protocol for a realist case study of the Ned App. JMIR Res Protoc.

[72] Taki S, Lymer S, Russell CG, Campbell K, Laws R, Ong KL, Elliott R, Denney-Wilson E (2017). Assessing user engagement of an mHealth intervention: development and implementation of the growing healthy app engagement index. JMIR Mhealth Uhealth.

[73] Thies K, Anderson D, Cramer B (2017). Lack of adoption of a mobile app to support patient self-management of diabetes and hypertension in a federally qualified health center: interview analysis of staff and patients in a failed randomized trial. JMIR Hum Factors.

[74] Toro-Ramos T, Kim Y, Wood M, Rajda J, Niejadlik K, Honcz J, Marrero D, Fawer A, Michaelides A (2017). Efficacy of a mobile hypertension prevention delivery platform with human coaching. J Hum Hypertens.

[75] Beiwinkel T, Hey S, Bock O, Rössler W (2017). Supportive mental health self-monitoring among smartphone users with psychological distress: protocol for a fully mobile randomized controlled trial. Front Public Health.

[76] Lei H, Nahum-Shani I, Lynch K, Oslin D, Murphy SA (2012). A "SMART" design for building individualized treatment sequences. Annu Rev Clin Psychol.

[77] Almirall D, Nahum-Shani I, Sherwood NE, Murphy SA (2014). Introduction to SMART designs for the development of adaptive interventions: with application to weight loss research. Transl Behav Med.

[78] Collins LM, Murphy SA, Strecher V (2007). The multiphase optimization strategy (MOST) and the sequential multiple assignment randomized trial (SMART): new methods for more potent eHealth interventions. Am J Prev Med.

[79] Serrano KJ, Yu M, Coa KI, Collins LM, Atienza AA (2016). Mining health app data to find more and less successful weight loss subgroups. J Med Internet Res.

[80] Morrison C, Doherty G (2014). Analyzing engagement in a web-based intervention platform through visualizing log-data. J Med Internet Res.

[81] Dennison L, Morrison L, Conway G, Yardley L (2013). Opportunities and challenges for smartphone applications in supporting health behavior change: qualitative study. J Med Internet Res.

[82] Morrison LG, Hargood C, Lin SX, Dennison L, Joseph J, Hughes S, Michaelides DT, Johnston D, Johnston M, Michie S, Little P, Smith PW, Weal MJ, Yardley L (2014). Understanding usage of a hybrid website and smartphone app for weight management: a mixed-methods study. J Med Internet Res.

[83] Morrison LG, Geraghty AW, Lloyd S, Goodman N, Michaelides DT, Hargood C, Weal M, Yardley L (2018). Comparing usage of a web and app stress management intervention: an observational study. Internet Interv.

[84] Stinson JN, Lalloo C, Harris L, Isaac L, Campbell F, Brown S, Ruskin D, Gordon A, Galonski M, Pink LR, Buckley N, Henry JL, White M, Karim A (2014). iCanCope with Pain™: user-centred design of a web- and mobile-based self-management program for youth with chronic pain based on identified health care needs. Pain Res Manag.

[85] Yardley L, Choudhury T, Patrick K, Michie S (2016). Current issues and future directions for research into digital behavior change interventions. Am J Prev Med.

[86] Patrick K, Hekler EB, Estrin D, Mohr DC, Riper H, Crane D, Godino J, Riley WT (2016). The pace of technologic change: implications for digital health behavior intervention research. Am J Prev Med.

[87] Scherer E, Ben-Zeev D, Li Z, Kane J (2017). Analyzing mHealth engagement: joint models for intensively collected user engagement data. JMIR Mhealth Uhealth.

[88] Tignor N, Wang P, Genes N, Rogers L, Hershman SG, Scott ER, Zweig M, Yvonne Chan YF, Schadt EE (2017). Methods for clustering time series data acquired from mobile health apps. Pac Symp Biocomput.

[89] Nilsen W (2015). American Association for the Advancement of Science.

[90] Kumar S, Nilsen WJ, Abernethy A, Atienza A, Patrick K, Pavel M, Riley WT, Shar A, Spring B, Spruijt-Metz D, Hedeker D, Honavar V, Kravitz R, Lefebvre RC, Mohr DC, Murphy SA, Quinn C, Shusterman V, Swendeman D (2013). Mobile health technology evaluation: the mHealth evidence workshop. Am J Prev Med.

[91] The Lancet (2018). Is digital medicine different?. Lancet.

[92] The National Institute for Health and Care Excellence.

