# Supplementary material for: A Library of Analytic Indicators to Evaluate Effective Engagement with Consumer mHealth Apps for Chronic Conditions: Scoping Review
Source: JMIR Mhealth Uhealth. 2019 Jan 18;7(1):e11941. doi: 10.2196/11941 (PMC6356188; doi:10.2196/11941)
Supplement: Multimedia Appendix 1 [file mhealth_v7i1e11941_app1.pdf]

Multimedia Appendix 1. Scoping review search strategy for MEDLINE on the Ovid platform.

1. engage*.mp,tw.
2. flow*.mp,tw.
3. exp Attention/ or attenti*.mp,tw.
4. interest*.mp,tw.
5. immers*.mp,tw.
6. focus*.mp,tw.
7. absor*.mp,tw.
8. affect.mp,tw. or exp Affect/
9. usage.mp,tw.
10. usability.mp,tw.
11. use*.mp,tw.
12. dose.mp,tw.
13. adherence.mp,tw. or exp Patient Compliance/
14. amount*.mp,tw.
15. breadth.mp,tw.
16. duration.mp,tw.
17. frequency.mp,tw.
18. depth.mp,tw.
19. stickiness.mp,tw.
20. lurk*.mp,tw.
21. disengage*.mp,tw.
22. attrition.mp,tw.
23. exp Patient Dropouts/ or dropout.mp,tw.
24. retention.mp,tw. or exp "Retention (Psychology)"/
25. experienc*.mp,tw.
26. pattern*.mp,tw.
27. trend*.mp,tw.
28. uptake.mp,tw.
29. feasib*.mp,tw.
30. efficac*.mp,tw.
31. effect*.mp,tw.
32. accept*.mp,tw.
33. adhere*.mp,tw.
34. 1 or 2 or 3 or 4 or 5 or 6 or 7 or 8 or 9 or 10 or 11 or 12 or 13 or 14 or 15 or 16 or 17 or 18 or 19 or 20 or 21 or 22 or 23 or 24 or 25 or 26 or 27 or 28 or 29 or 30 or 31 or 32 or 33
35. mobile health.tw.
36. mHealth.tw.
37. m-health.tw.
38. smartphone app*.tw.
39. 35 or 36 or 37 or 38
40. evaluation.mp. or exp Evaluation Studies as Topic/
41. evaluat*.tw.
42. study.tw.
43. trial.tw.
44. assess*.tw.
45. exp Clinical Trial/
46. 40 or 41 or 42 or 43 or 44 or 45
47. 34 and 39 and 46
48. limit 47 to (abstracts and english language and yr="2015 - 2017")
