# Supplementary material for: A Library of Analytic Indicators to Evaluate Effective Engagement with Consumer mHealth Apps for Chronic Conditions: Scoping Review
Source: JMIR Mhealth Uhealth. 2019 Jan 18;7(1):e11941. doi: 10.2196/11941 (PMC6356188; doi:10.2196/11941)
Supplement: Multimedia Appendix 3 [file mhealth_v7i1e11941_app3.pdf]

Multimedia Appendix 3. Practical considerations for applying analytic indicators to evaluate effective engagement.

Suppose we were evaluating effective engagement with an mHealth app for panic disorder self-management [1], and wanted to establish a digital dose-response relationship between (1) the number of digitally-mediated breathing exercises performed, and (2) panic disorder severity. Suppose also that users were asked to self-report their panic disorder severity on a daily basis through an in-app implementation of the validated Panic Disorder Severity Scale (PDSS) [2]. Our first task will be to insert an analytic tag into the app's source code to denote that selecting the 'Done' button at the end of a breathing exercise signifies that it has been performed. Each time a user selects this button, an analytic event will be logged and stored alongside a user identifier and timestamp. We will also insert a different analytic tag to the 'Close' button at the end of a PDSS survey. Thus, when a user reports their panic disorder severity and closes the survey, another analytic event will be logged and stored with the survey score, user identifier, and timestamp. We can now query our two analytic data fields and analyze the stored data points to assess the relationship between the number of digitally-mediated breathing exercises performed and panic disorder severity. The inclusion of a user identifier in the metadata of both events enables linking them to explore a dose-dependent relationship, and consequently determine how many breathing exercises should be completed to experience a significant decrease in panic disorder severity.

References

1. Pham Q, Khatib Y, Stansfeld S, Fox S, Green T. Feasibility and Efficacy of an mHealth Game for Managing Anxiety: "Flowy" Randomized Controlled Pilot Trial and Design Evaluation. Games Health J [Internet]. 2016 Feb 4 [cited 2015 Dec 21];5(1):50–67. Available from: http://online.liebertpub.com/doi/10.1089/g4h.2015.0033
2. Houck PR, Spiegel DA, Shear MK, Rucci P. Reliability of the self-report version of the panic disorder severity scale. Depress Anxiety [Internet]. 2002 [cited 2018 Jul 21];15(4):183–5. Available from: http://www.ncbi.nlm.nih.gov/pubmed/12112724
